# Quantitative trait loci associated with different polar metabolites in perennial ryegrass - providing scope for breeding towards increasing certain polar metabolites

**DOI:** 10.1186/s12863-017-0552-0

**Published:** 2017-10-10

**Authors:** Alexandre Foito, Christine Anne Hackett, Derek Stewart, Janaki Velmurugan, Dan Milbourne, Stephen L. Byrne, Susanne Barth

**Affiliations:** 1 Teagasc, Crops Environment and Land Use Programme, Oak Park Research Centre, Carlow, Ireland; 2 Enhancing Crop Productivity and Utilisation, The James Hutton Institute, Invergowrie, Dundee, DD2 5DA UK; 3 Biomathematics and Statistics Scotland, Invergowrie, Dundee, DD2 5DA UK; 4 Engineering and Physical Sciences, Heriot-Watt University, Edinburgh, EH14 4AS UK; 5 Norwegian Institute of Bioeconomy Research, Pb 115, -1431 Ås, NO Norway

**Keywords:** Perennial ryegrass, *Lolium perenne*, QTL, Polar metabolites, Aminoacids, Soluble carbohydrates, Myo-inositol, Citric acid and 2, 3-hydroxypropanoic acid

## Abstract

**Background:**

Recent advances in the mapping of biochemical traits have been reported in *Lolium perenne*. Although the mapped traits, including individual sugars and fatty acids, contribute greatly towards ruminant productivity, organic acids and amino acids have been largely understudied despite their influence on the ruminal microbiome.

**Results:**

In this study, we used a targeted gas-chromatography mass spectrometry (GC-MS) approach to profile the levels of 25 polar metabolites from different classes (sugars, amino acids, phenolic acids, organic acids and other nitrogen-containing compounds) present in a *L. perenne* F2 population consisting of 325 individuals. A quantitative trait (QTL) mapping approach was applied and successfully identified QTLs regulating seven of those polar metabolites (L-serine, L-leucine, glucose, fructose, myo-inositol, citric acid and 2, 3-hydroxypropanoic acid).Two QTL mapping approaches were carried out using SNP markers on about half of the population only and an imputation approach using SNP and DArT markers on the entire population. The imputation approach confirmed the four QTLs found in the SNP-only analysis and identified a further seven QTLs.

**Conclusions:**

These results highlight the potential of utilising molecular assisted breeding in perennial ryegrass to modulate a range of biochemical quality traits with downstream effects in livestock productivity and ruminal digestion.

**Electronic supplementary material:**

The online version of this article (10.1186/s12863-017-0552-0) contains supplementary material, which is available to authorized users.

## Background

Perennial ryegrass (*Lolium perenne*) is one of the major species present in temperate grasslands and its use as forage is essential for the sustainable diets of grazing animals which impact dairy and meat production. Consequently, there is a large interest in breeding new varieties of ryegrass with improved yield, quality and disease resistance, but also improved downstream effects in meat and milk composition [1–3]. Recent developments in the field of genetical genomics, allied with developments in high throughput trait screening [4] are likely to accelerate the development of new varieties in comparison with traditional breeding. In fact, a number of perennial ryegrass mapping populations have been developed over time and probed using different molecular marker systems (e.g. [3, 5–7]. Due to the importance of some components in the diet of ruminants, a number of studies have been reported with the goal of identifying linkage regions responsible for the levels of some traits such as individual sugars [8], crude protein content [9, 10], fatty acid and non-polar composition [3, 11]. Indeed, some, if not all, of these traits are likely to be involved in animal productivity which can be illustrated by several studies. For example, the consumption of a perennial ryegrass variety with enhanced water-soluble carbohydrate (WSC) levels significantly increased live weight gain of suckling lambs compared to varieties with lower WSC levels [12]. In addition, a meta-analysis highlighted that crude protein content is the most important dietary factor influencing milk protein synthesis in dairy cows [13].

However, studies aiming to map key basal nutrient components have been largely confined to protein content and sugar and fructan composition, with very little attention devoted to polar small molecules including important classes of compounds such as individual amino acids and organic acids. Indeed, from a nutritional perspective, a major function of proteins is to supply adequate amounts of required “essential” amino acids (EAA) [14] which cannot be biosynthesized de novo by animals and need to be present in their diets and often supplemented in livestock feed. This highlights that animal productivity is not only determined by the levels of crude protein but also by the quality of the consumed protein particularly with respect to EAA composition. However, free amino acids interact with ruminal bacterial and protozoa communities experiencing extensive degradation and modification [15]. Therefore, although the levels of free amino acids in the diets of ruminants are unlikely to be nutritionally relevant, its presence could have an important effect at microbiome level [16]. Interestingly, it has been proposed that the levels of organic acids, such as malate, present in ruminant diets could have implications to the rumen microbiome, and has been proposed as a strategy for the management of ruminant acidosis [17, 18]. Considering the phenolic content, there is an argument for and against their presence in ryegrass. The downside is their negative impact on rye grass digestibility [19]. Conversely, studies in buckwheat identified that the phenolics reduced methane emission during rumen digestion.

Clearly, a number of different classes of polar compounds such as sugars and amino acids have an important role in animal nutrition and productivity. In this study, we used a targeted gas-chromatography mass spectrometry (GC-MS) approach to profile the levels of 25 polar metabolites from different classes (sugars, amino acids, phenolic acids, organic acids and other nitrogen-containing compounds) present in a *L. perenne* F2 population. We previously investigated quantitative trait loci (QTLs) for the non-polar metabolites for this population using a Diversity Arrays Technology marker (DArT)-based map [11]. The objectives of the current study was to compare the efficacy of two mapping approaches, mapping with a denser SNP map in a subset of the F2 population and mapping with a combined SNP and DArT map in the full population to test for QTLs for polar metabolites, several of which had not been previously mapped or reported on in perennial ryegrass.

## Methods

### Plant material and experimental design

The F2 progeny consisting of 325 genotypes was generated from the cross of two inbred *Lolium* lines as described by [8]. The trial was laid out as an alpha lattice design with two replicates in the field. Each replicate was comprised of 45 incomplete blocks which contained nine genotypes with one replicate of either the maternal, paternal or F1 genotype. The experiment was planted in a free-draining grey brown podzolic soil in Oak Park, Carlow, Ireland (52.861° N, 6.915° W; 58 m above sea level) in the spring of 2006 in mini swards containing six clonal replicates of a single genotype each.

### Sampling and extraction

Plants were sampled for metabolite profiling in August 2007 within the same day as described in [11]. In summary, plants were cut at the height of 6 cm and the leaf blades were transferred into Eppendorf microcentrifuge tubes (2 mL) and immediately transferred into dry-ice before being cooled with liquid nitrogen and stored at −80 °C until lyophilization. Once lyophilised the freeze-dried material was homogenized and approximately 12 mg were extracted sequentially in methanol (3 mL), water (1.5 mL), chloroform (6 mL) and water (750 μL) at 30 °C for 30 min each incubation. During the initial extraction step, 100 μL ribitol (2 mg mL^−1^) was added as an internal standard. At the end of the sequential extraction samples were centrifuged for 10 mins and a biphasic system was generated. The top fraction, corresponding to a polar fraction, was isolated and transferred into glass vials.

### Derivatisation of polar and non-polar extracts for GC-MS analysis

The analysis of the GC-MS derivatised polar fractions was performed as described in [20]. In summary, an aliquot of the polar fraction (250 μL) was evaporated at reduced pressure and methoxymated using 80 μL methoxylamine hydrochloride solution (20 mg mL^−1^ prepared in pyridine). Samples were subsequently silylated using 80 μL of N-methyl-N-(trimethylsilyl)-trifluroacetamide (MSTFA). A sub-sample (40 μL) of the derivatised extract was added to an autosampler vial containing 50 μL of a dried retention standard mixture (undecane, tridecane, hexadecane, eicosane, tetracosane, triacontane, tetratriacontane and octatriacontane at a concentration of 0.2 mg mL-1 in isohexane) and diluted with pyridine (1:1) prior analysis by GC-MS with the settings described in [11].

### Analysis of polar fractions by GC-MS

The acquisition of GC-MS profiles was performed on a Thermo DSQ system (Thermo-Finnigan, UK) using the settings described in [20]. The samples were run across 18 batches for each fraction (polar and non-polar), with the first replicate experiment of the experimental design being completely analysed first (two technical replicates), followed by the analysis of the second replicate (one technical replicate). Each batch analysed included a minimum of three biological replicates from the paternal, maternal or F1 genotypes. A targeted analysis allowed the semi-quantification of 25 polar metabolites (Additional file 1: Table S1) by normalisation to the internal standard as described in [20]. For most cases, identity assignment was based on comparison of predominant unique mass and kovat retention index with previously analysed authentic standards (purchased from Sigma-Aldrich, Dorset, UK). Alternatively, compounds were tentatively annotated based on comparison with GC-MS database NIST/EPA/NIH mass spectral library NIST version 08 (for more details on each compound see Additional file 1: Table S1). Quality control across the different batches was monitored by the analysis of external reference material which consisted of freeze-dried tuber material from *Solanum tuberosum* (cv. ‘Desiree’) extracted and derivatised as described above.

Principal component analysis (PCA) was used to summarise broad scale variation among the ryegrass samples using the metabolites in Additional file 1: Table S1. For metabolomics metabolite profiling data, PCA can be carried out using either the sample variance-covariance matrix or the sample correlation matrix: The former focuses on the most abundant metabolites, whereas the latter standardises each metabolite by subtracting the mean and dividing by the standard deviation. Both approaches were investigated here to see which was the most informative. The first PCA was based on the ryegrass generations with the highest replication e.g. the parents and F1. A second PCA was then used to explore the relationships between the ryegrass parents, the F1 and the F2 lines.

A mixed model was used to estimate genotype means for the parents, F1 and the F2 lines, including random effects of block and plot in the field trial and the batches and sequence position in the GC-MS analysis. The generation was treated as a fixed effect and the genotypes within the F2 generation were fitted as random effects to estimate the heritability of the metabolites, and then as fixed effects to estimate the F2 genotype means for QTL analysis. The analysis was performed on log-transformed data to stabilise the variance. The model and the two estimators of heritability ${H}_A^2$ and ${H}_P^2$ are the same as in the models used for the non-polar metabolites from this population by [11]. Two estimators of heritability were compared for this experiment. *H*_*A*_^*2*^ is a natural extension of the usual estimator:

1 $$ {H}_A^2=\frac{\sigma_G^2}{\sigma_G^2+{\sigma}_P^2+{\sigma}_S^2} $$

where *σ*_*P*_^*2*^ is the variation due to field plots and *σ*_*S*_^*2*^ is the variation due to the laboratory sequence. An alternative method is given by Holland et al. [21] and by Piepho and Möhring [22]:

2 $$ {H}_P^2=\frac{\sigma_G^2}{\sigma_G^2+\overline{v}/2} $$

where *v̅* is the mean variance of a difference of two adjusted genotype means (BLUE) from REML.

The mixed model was fitted again, with the offspring term *f* fitted as a fixed effect, to estimate the offspring genotype means for QTL analysis.

### Linkage map and QTL analysis

SNP data was collected using genotyping-by-sequencing (GbS) from a subset of 149 genotypes of this F2 population (DNA for these 149 individuals had been readily available at the time of GbS work) and a linkage map was constructed using Joinmap 4.1 [23, 24] in a similar approach as described by [7]. For SNP alignments the perennial ryegrass genome by [25] was used. SNPs with more than 10% missing values were excluded. The following filters were applied on the SNP calls to select markers for constructing the genetic map. First, the markers with the genotype quality of (phred <30) were recorded as missing. Second, markers with 90% of individuals genotyped and that are heterozygous in the F1 were used as filters. Applying the above filtering criteria yielded 4253 markers for 151 individuals. The filtered markers were then imported into R/qtl [26] using *‘read.cross’* function and the pairwise recombinant fractions (RF) were estimated using *‘est.rf’* function. The markers were grouped using *‘formLinkageGroups’* function and at RF/LOD threshold of 0.15/30, the markers grouped into big 14 linkage groups and few small groups. The heat map obtained using the *‘plot.RF’* function showed that markers in some of the 14 groups had switched alleles. After switching the alleles using *‘switchAlleles’* function in the following groups 4, 6, 7, 10, 12, 13, 14, the pairwise recombinant fractions were estimated again using *‘est.rf’* function and grouped using *‘formLinkageGroups’* function. At RF/LOD threshold of 0.15/30, this resulted in 7 big linkage groups with 3953 markers. Only one marker per scaffold was retained in the final map for QTL mapping. Summary statistics for the SNP map are given in Table 1.

**Table 1:** Composition of the F2 genetic map grouped by linkage group (LG) and detailed by number of SNP markers, number of non-redundant SNP markers, map lengths (in cM), number of SNP markers per scaffold, map length for each of the seven chromosomes (based on multiple SNPs and 1 SNP per scaffold) and number of DArT markers added per chromosome

LG	Full map			Map with one marker per scaffold		DArT markers
	SNP markers	non-redundant SNPs	map length (cM)	SNP markers	map length (cM)	
1	474	230	110.2	136	99.7	50
2	594	299	138.9	183	127.2	42
3	703	378	155.6	209	119.8	60
4	668	353	147.7	221	127.9	60
5	514	325	137.4	162	112.4	43
6	437	214	100.9	134	90.1	26
7	563	286	126.9	166	106.0	45
Total	3953	2085	917.6	1211	783.0	326

Two different QTL analyses were carried out. The first analysis used only the 149 out of the total of 325 F2 genotypes for which SNP data was available. QTL mapping was carried out for these 149 genotypes based the GBS SNP map, and using interval mapping with the MapQTL 5 software [23]. A permutation test with 1000 permutations was used to establish a 5% genome-wide threshold as LOD = 3.5 (10% threshold = 3.1). Restricted multiple QTL mapping (rMQM) in MapQTL 5 was used to search for additional QTLs, using the selected QTLs as cofactors. All phenotypic and genetic mapping data are available in Additional file 2.

As this approach uses less than half of the population, an alternative analysis was used where complete genotypes were simulated for the remaining F2 genotypes conditional on the observed SNP data in conjunction with the DArT information from [11]. The DArT markers were placed onto the SNP map between the SNPs with which they had the strongest association, based on a chi-square test of independence. Table 1 shows the numbers of DArT markers added to each linkage group. The function ‘sim.geno’ in the R/qtl package (version 1.39–5) [26] was used to simulate complete genotype data on a 1 cM grid of positions along each chromosome, conditional on the observed SNP and DArT markers. One hundred complete datasets were simulated. QTL interval mapping was carried out using the R/qtl function ‘scanone’ with method = Imputation, and multiple QTL models were fitted with the ‘fitqtl’ function. The ‘scanone’ function forms a combined LOD score over the 100 simulated datasets as a trimmed mean (excluding the three highest and three lowest) on the 10^LOD^ scale [27]. A permutation test with 1000 permutations indicated that a 5% genome-wide threshold of LOD = 3.3 (10% threshold 3.0) should be used for this analysis.

## Results

### Exploratory analysis of the parents and F1 generation

Using the log-transformed concentration of all the 25 polar metabolites, a phenotypic analysis was performed to identify significant effects among the parents and F1 offspring. The results are summarised in Table 2. The mean metabolite levels were significantly different (*p* < 0.05) amongst the paternal, maternal and F1 lines for 19 of the 25 metabolites analysed. By far the most significant metabolite was inositol (Table 2). For *myo*-inositol the paternal line was found to have a higher mean compared with the F1 population and the maternal line. Additional file 3: Figure S1 shows a plot of the first two principal components, based on the correlation matrix, which explain 21.5 and 16.6% of the overall variation in the metabolite profiles of the parental and F1 lines. The maternal line was generally separated from the other lines on the first principal component (PC1), which showed high positive loadings for L-glycine, L-glutamate, L-threonine, *myo*-inositol, 2,3-dihydroxypropanoic acid, L-serine, γ–aminobutyric acid (GABA) and threonic acid whereas negative loadings were found for fructose, glucose and galactose. The F1 and the parental lines were partially separated by the second principal component (PC2), which had high positive loadings for proline and glucose, and negative loadings for L-aspartate.

**Table 2:** Summary statistics for the log-transformed data of the polar metabolites in the perennial ryegrass population

	Metabolites	P_f_mean	P_m_mean	F1_mean	*a*	*d*	F	P_f_P_m_sed	P_f_F1_sed	P_m_F1_sed
1	L-leucine	−5.39	−5.70	−5.39	0.16	0.16	0.94	0.261	0.238	0.253
2	Glycerol	−4.23	−4.08	−4.05	−0.08	0.11	1.39	0.128	0.116	0.125
3	L-isoleucine	−5.49	−5.56	−5.64	0.03	−0.11	0.85	0.126	0.114	0.123
4	L-proline	−4.63	−4.31	−5.61	−0.16	−1.13	11.96^‡***^	0.296	0.273	0.284
5	L-glycine	−4.07	−5.20	−4.32	0.56	0.31	24.80^***^	0.166	0.152	0.161
6	2,3-dihydroxypropanoic acid	−4.80	−5.14	−4.64	0.17	0.33	5.85^**^	0.152	0.139	0.147
7	L-serine	−3.29	−4.17	−3.58	0.44	0.14	27.27^***^	0.120	0.108	0.118
8	L-threonine	−5.08	−5.56	−5.36	0.24	−0.04	6.40^**^	0.139	0.125	0.136
9	Malic acid	−3.82	−4.38	−2.96	0.28	1.14	15.96^***^	0.271	0.234	0.263
10	L-aspartate	−5.36	−5.62	−4.64	0.13	0.85	14.09^***^	0.210	0.190	0.198
11	γ-amino butyric acid	−2.69	−3.78	−3.10	0.55	0.14	16.53^***^	0.190	0.171	0.186
12	Threonic acid	−5.02	−6.06	−5.18	0.52	0.36	11.62^***^	0.234	0.217	0.222
13	L-glutamate	−4.62	−5.29	−4.43	0.33	0.52	7.60^**^	0.231	0.204	0.223
14	Putrescine	−4.89	−5.06	−6.02	0.09	−1.05	14.83^***^	0.247	0.223	0.242
15	Citric acid	−5.31	−6.06	−4.11	0.37	1.57	23.28^***^	0.307	0.278	0.293
16	Shikimic acid	−6.03	−6.20	−6.53	0.08	−0.41	1.17	0.361	0.327	0.352
17	Galactose	−6.79	−6.10	−6.93	−0.34	−0.49	7.58^**^	0.229	0.209	0.222
18	L-lysine	−8.34	−9.09	−9.28	0.37	−0.56	1.91	0.559	0.498	0.548
19	L-tyrosine	−6.46	−5.93	−6.43	−0.27	−0.24	2.11	0.289	0.261	0.280
20	*myo*-Inositol	−2.58	−3.71	−3.62	0.57	−0.47	45.68^***^	0.136	0.125	0.130
21	Glucose/Galactose-glycerol conjugate	−4.72	−4.84	−4.78	0.06	0.00	0.10	0.276	0.253	0.265
22	Sucrose	1.71	1.16	1.61	0.28	0.18	15.40^***^	0.105	0.096	0.102
23	Chlorogenic acid	−6.81	−9.72	−9.07	1.46	−0.80	6.63^**^	0.876	0.786	0.858
24	Fructose	−2.02	−1.18	−2.12	−0.42	−0.51	26.60^***^	0.141	0.130	0.136
25	Glucose	−1.27	−0.92	−1.59	−0.18	−0.49	18.02^***^	0.115	0.104	0.112

***=*p* < 0.001; **=*p* < 0.01; *=*p* < 0.05; s.e.d.: standard error of difference; ns: not-significant; P_f_mean, P_m_mean, F1_mean are the means of the paternal parent, maternal parent and F1 respectively and *a* and *d* are the estimated net additive and dominance effects. F is the F-statistic testing for significance among the generation means, and the standard errors of difference between pairs of means are in the last three columns

### Estimation of heritability

Estimates of the variance components and the heritabilities of the polar components from the mixed model analysis of the full population are shown in Table 3. *Myo*-inositol which had the highest heritability (${H}_A^2$ and ${H}_P^2$of 34.1% and 30.2%, respectively) was also found to have the most significant QTL effect (see Tables 5 and 6). Citric acid, fructose, glucose and sucrose also had relatively high heritabilities (> 20%) and QTLs were detected (see below) for all of these except sucrose.

**Table 3:** Variance components and heritabilities of the polar components

	Metabolites	${H}_A^2$	${H}_P^2$	${\sigma}_R^2$	${\sigma}_P^2$	${\sigma}_I^2$	${\sigma}_B^2$	${\sigma}_S^2$	${\sigma}_G^2$	*v̅*
1	L-leucine‡	2.83	4.00	0.007	0.529	0.000	0.174	0.190	0.021 ns	1.005
2	Glycerol	10.17	13.27	0.000	0.147	0.000	0.062	0.039	0.021 ns	0.275
3	L-isoleucine	15.91	19.02	0.035	0.526	0.000	0.133	0.179	0.133**	1.135
4	L-proline	6.54	8.48	0.040	1.155	0.000	0.082	0.161	0.092 ns	1.987
5	L-glycine	11.61	13.59	0.000	0.262	0.027	0.152	0.043	0.040 ns	0.509
6	2,3-dihydroxypropanoic acid ‡	17.00	21.87	0.025	0.163	0.000	0.069	0.077	0.049**	0.351
7	L-serine‡	19.17	22.22	0.000	0.202	0.014	0.090	0.038	0.057**	0.399
8	L-threonine	5.54	7.46	0.000	0.199	0.001	0.086	0.169	0.022 ns	0.535
9	Malic acid	7.62	7.87	0.000	0.467	0.068	0.357	0.015	0.040 ns	0.930
10	L-aspartate	14.40	14.26	0.012	0.477	0.063	0.137	0.014	0.083 ns	0.994
11	γ-amino butyric acid	15.81	12.22	0.000	0.116	0.276	0.238	0.095	0.040 ns	0.569
12	Threonic acid	0.68	0.94	0.000	0.479	0.089	0.020	0.000	0.003 ns	0.695
13	L-glutamate	18.51	19.54	0.000	0.355	0.062	0.696	0.030	0.087 ns	0.720
14	Putrescine	18.97	19.55	0.004	0.529	0.254	0.451	0.197	0.170*	1.400
15	Citric acid‡	31.02	27.08	0.001	0.493	0.241	0.310	0.017	0.229**	1.236
16	Shikimic acid	5.66	4.88	0.000	0.500	0.472	0.448	0.284	0.047 ns	1.831
17	Galactose	16.78	17.12	0.000	0.173	0.200	0.138	0.234	0.082*	0.794
18	L-lysine	0.67	1.09	0.000	0.352	0.000	2.046	5.711	0.041 ns	7.415
19	L-tyrosine	1.88	2.06	0.020	0.303	0.211	0.206	0.293	0.011 ns	1.086
20	Myo-inositol‡	34.10	30.25	0.000	0.057	0.074	0.011	0.021	0.0405**	0.187
21	Glucose/Galactose-glycerol conjugate	2.42	3.19	0.080	0.592	0.040	0.128	0.079	0.0166 ns	1.008
22	Sucrose	20.02	25.38	0.002	0.120	0.000	0.051	0.016	0.0338**	0.199
23	Chlorogenic acid	0.70	1.16	0.707	2.026	0.000	6.801	7.062	0.0642 ns	10.960
24	Fructose‡	26.00	32.03	0.000	0.158	0.000	0.027	0.030	0.0663***	0.281
25	Glucose‡	26.55	32.99	0.007	0.140	0.000	0.109	0.017	0.0567***	0.230

***=*p* < 0.001; **=*p* < 0.01; *=*p* < 0.05; ns: not-significant; ${H}_A^2$ and${H}_P^2$denote different measures of heritability, as estimated from eqs. (1) and (2) of Foito et al. (2015) respectively. Variance components due to field replicate (${\sigma}_R^2$), plot (${\sigma}_P^2$), technical replication (${\sigma}_I^2$), analysis batch (${\sigma}_B^2$), sequence order (${\sigma}_S^2$) and offspring (${\sigma}_G^2$) are represented. *v̅* is the mean variance of a difference of two genotype means. The significance of the offspring component, as tested by the change in deviance, is included. A double dagger (‡) shows metabolites for which a QTL was detected

### Correlation among metabolites

A metabolite-metabolite correlation analysis based on the log-scale means of the F2 lines is represented in Table 4. All the significant correlations were positive with the strongest correlation found between glucose and fructose (0.83). The compound with the most number of significant correlations was L-serine which had positive correlations with L-glycine, L-aspartate and GABA (0.60, 0.52 and 0.54, respectively).

**Table 4:** Correlation matrix for the metabolites based on the F2 genotype means on a log scale

	Metabolite	1	2	3	4	5	6	7	8	9	10	11	12	13	14	15	16	17	18	19	20	21	22	23	24
1	L-leucine																								
2	Glycerol	0.17																							
3	L-isoleucine	0.40	0.19																						
4	L-proline	−0.10	−0.08	0.08																					
5	Glycine	0.24	0.06	0.17	−0.07																				
6	2,3-dihydroxypropanoic acid acidGlyceric acidGlyceric acid	0.19	0.18	0.03	−0.16	0.39																			
7	L-serine	0.24	0.13	0.23	0.10	0.60	0.41																		
8	L-threonine	0.01	0.04	0.08	0.17	0.34	0.11	0.53																	
9	Malic acid	0.20	0.14	0.18	−0.06	0.32	0.47	0.42	0.11																
10	L-aspartate	0.06	0.12	0.20	0.10	0.33	0.14	0.52	0.23	0.39															
11	γ-amino butyric acid	0.10	0.16	0.19	0.06	0.30	0.23	0.54	0.39	0.25	0.37														
12	Threonic acid	0.00	0.02	0.09	−0.01	0.33	0.30	0.36	0.30	0.25	0.23	0.44													
13	L-glutamate	0.11	0.03	−0.05	−0.10	0.41	0.38	0.38	0.16	0.46	0.35	0.12	0.25												
14	Putrescine	0.07	−0.11	0.03	0.20	0.05	−0.13	−0.09	−0.02	−0.22	−0.12	0.03	0.09	−0.05											
15	Citric acid	0.14	0.05	0.12	−0.17	0.23	0.30	0.29	0.10	0.59	0.39	0.23	0.27	0.56	−0.08										
16	Shikimic acid	−0.06	0.05	−0.06	−0.03	−0.04	0.00	0.05	0.03	0.12	0.05	0.06	0.16	0.17	0.02	0.07									
17	Galactose	0.08	0.16	0.05	0.18	0.00	−0.04	0.01	−0.05	−0.01	−0.03	−0.01	−0.05	−0.06	0.05	−0.09	0.03								
18	L-lysine	0.05	−0.05	−0.04	−0.07	−0.03	−0.12	−0.08	0.01	−0.09	−0.05	−0.08	0.07	0.07	0.21	0.01	0.14	0.08							
19	L-tyrosine	−0.01	0.06	−0.04	0.02	−0.06	−0.08	−0.10	0.00	−0.08	−0.11	0.00	−0.08	−0.06	0.01	−0.04	−0.10	−0.10	0.13						
20	Myo-inositol	−0.03	0.15	0.09	0.03	0.21	0.12	0.25	0.21	0.17	0.07	0.33	0.33	0.19	0.01	0.12	0.11	0.14	−0.03	0.05					
21	Glucose/galactose-glycerol conjugate	0.04	0.45	0.12	−0.01	0.01	0.24	0.14	−0.01	0.15	0.06	0.03	−0.07	0.13	−0.17	0.12	0.16	0.27	0.05	0.00	0.17				
22	Sucrose	−0.17	−0.15	−0.12	−0.03	0.17	0.18	0.04	−0.05	0.03	0.05	0.16	0.28	0.17	0.32	0.10	0.12	−0.09	−0.06	−0.10	0.25	−0.18			
23	Chlorogenic acid	−0.19	−0.09	0.04	0.02	0.01	−0.01	−0.01	0.02	−0.03	0.08	0.19	0.15	0.15	0.22	0.21	0.10	−0.05	0.05	0.02	0.18	−0.14	0.32		
24	Fructose	−0.07	0.20	0.02	0.22	−0.10	−0.14	−0.15	−0.09	−0.07	−0.03	0.07	0.03	−0.17	0.13	−0.08	0.13	0.48	0.11	0.13	0.26	0.07	0.00	0.11	
25	Glucose	−0.07	0.30	0.12	0.24	−0.05	−0.08	−0.12	−0.05	−0.09	−0.04	0.11	0.05	−0.14	0.26	−0.13	0.07	0.46	0.10	0.06	0.26	0.12	0.14	0.15	0.83

Correlations >0.5 are highlighted

### QTL detection

The SNP genetic linkage map used for QTL analysis had a map length of 783 cM and contained 1211 markers (Table 1). A further 326 DArT markers were placed on this map between the SNPs with which they had the strongest association, as detailed in Table 1. There was a high level of agreement between the DArT ordering from this approach and that in the DArT-only map of [11].

There was good agreement in position between the QTLs detected using the SNP-only map on the population subset and those detected using the combined SNP and DArT map for most metabolites (Tables 5 and 6). QTLs were detected by both methods for L-leucine (LG1), L-serine (LG3), *myo*-inositol (LG2) and fructose (LG3). The analysis of the full population, imputing the missing SNP genotype data from the DArT genotypes, found additional QTLs for *myo*-inositol (LG1), fructose and glucose (at the same position on LG7), citric acid (LG7) and 2, 3 -dihydroxypropanoic acid (LG1, LG2 and LG4). The QTL positions, estimated effects and the %variance explained are summarised in Tables 5 and 6. The one-LOD support intervals for the QTL locations found by the two approaches are generally very similar. The most significant QTLs detected were found for *myo*-inositol (LG2: LOD 8.85) and for fructose (LG3: LOD 7.91) which was highly correlated with glucose (0.83) and also the QTL co-localized on LG7. The two QTL for *myo*-inositol on LG1 and LG2 accounted together for 16% of the variation in the population.

**Table 5:** Identification of quantitative trait loci (QTL) polar components by interval and rMQM mapping, using the SNP map only

Trait	Group	Pos. (cM)	Marker	LOD	μ_A_	μ_H_	μ_B_	% Expl.	Add	se_Add	Dom	se_Dom	One-lod interval
L-leucine	LG 1	7.9	s16737r0011889_11077	4.99	0.004	0.004	0.002	15.00	0.35	0.100	0.39	0.132	4.5–13.3
Myo-inositol	LG 2	74.4	s36r0030222_14689	8.39	0.047	0.031	0.030	24.00	0.23	0.039	−0.17	0.052	65.2–78.8
Fructose	LG 3	57.3	s15405r0015202_5350	3.57	0.120	0.134	0.177	11.00	−0.20	0.054	−0.08	0.075	54.5–70.8
L-serine	LG 3	74.7	s3460r0022619_71110	5.11	0.035	0.024	0.021	15.40	0.25	0.051	−0.14	0.068	67.8–93.4

For each trait the linkage group, position on genetic map and nearest genetic marker on genetic map, LOD scores, means of the three genotype classes (μ_A_, μ_H_ and μ_B_), percentage of variation explained and additive and dominance effects with standard errors have been given. The additive and dominance effects and standard errors come from the analysis of the log-transformed data, but the genotype means have been back-transformed to the natural scale

**Table 6:** Identification of quantitative trait loci (QTL) polar components by mapping using SNP and DArT data in the full population (*N* = 325), with simulation of missing genotype data

Trait	Group	Pos. (cM)	Marker	LOD	μ_A_	μ_H_	μ_B_	% Expl.	Add	se_Add	Dom	se_Dom	One-lod interval
L-leucine	LG 1	8.0	s16737r0011889_11077	4.56	0.004	0.004	0.002	6.57	0.29	0.071	0.19	0.102	6–12
2,3-dihydroxypropanoic acid	LG 1	4.6	s893r0038731_60419	3.91	0.011	0.009	0.008	4.49	0.14	0.036	0.03	0.051	0–9
	LG 2	126.9	s7455r0038980_4917	4.53	0.010	0.010	0.007	6.48	0.09	0.035	0.21	0.052	120–127
	LG 4	125.9	s1679r0036123_7554	3.51	0.008	0.009	0.011	5.05	−0.15	0.036	−0.02	0.063	108–128
Myo-inositol	LG 1	36.9	s697r0011191_46705	3.37	0.037	0.034	0.030	4.55	0.08	0.028	0.11	0.041	23–60
	LG 2	65.6	s13073r0029219_7352	8.85	0.047	0.032	0.030	12.05	0.17	0.031	−0.17	0.041	65–75
Fructose	LG 3	57.3	s15405r0015202_5350	7.91	0.119	0.135	0.178	11.82	−0.21	0.035	−0.06	0.049	55–66
	LG 7	36.1	rv1411	3.98	0.163	0.139	0.125	6.42	0.14	0.035	−0.12	0.048	29–48
Glucose	LG 7	36.1	rv1411	4.47	0.245	0.207	0.174	6.48	0.15	0.035	−0.09	0.049	31–48
L-serine	LG 3	71.6	s13354r0039793_22015	3.53	0.035	0.024	0.021	5.16	0.15	0.037	−0.05	0.053	37–91
Citric acid	LG 7	72.8	s15061r0016601_15890	3.39	0.014	0.012	0.008	5.49	0.30	0.080	0.14	0.112	38–95

For each trait the linkage group, position on genetic map and nearest genetic marker on genetic map, LOD scores, means of the three genotype classes (μ_A_, μ_H_ and μ_B_), percentage of variation explained and additive and dominance effects with standard errors have been given. The additive and dominance effects and standard errors come from the analysis of the log-transformed data, but the genotype means have been back-transformed to the natural scale

## Discussion

### Exploratory analysis of the parents and F1 generation

The results of our study reflect a diverse phytochemical composition present among the parental lines and the F1 lines that was exploited using a QTL mapping approach. The PCA plots of the complete dataset including all parental, F1 and F2 lines also demonstrated a separation between maternal and paternal lines (Additional file 4: Figure S2).

### Correlation among metabolites

The significant correlations were positive with the strongest correlation found between glucose and fructose (0.83), which is unsurprising due to their close metabolic pathways. The correlation between L-serine and L-glycine could result from their close relationship in the mitochondrial reactions of photorespiratory cycle mediated by the glycine decarboxylase/serine hydroxymethyl transferase complex (EC 2.1.2.1). The levels of citric acid correlated with the levels of L-glutamate (0.56) which can be explained by the relative close metabolic pathway and the dependence of glutamate synthesis from intermediates from the first part of the citric acid cycle.

### QTLs for myo-inositol accumulation on LGs 1 and 2

*myo*-Inositol and its phosphorylated derivatives have a variety of roles in cells that include stress response [28, 29] and secondary signal transduction [30, 31]. Indeed, the aspects of accumulation of myo-inositol during stress responses have been previously described in various plant species [32–34], including *Lolium perenne* [20]. Indeed, a perennial ryegrass genotype that displayed improved tolerance to a water-induced stress was found to accumulate increased levels of several compounds including myo-inositol and fructose (the latter with a QTL on LG3 and LG7), compared to the control conditions [20]. This clearly highlights the potential of improving the tolerance of perennial ryegrass to a range of different abiotic stresses by selecting genetic markers that are involved in the control of the levels of fructose and *myo*-inositol.

### Genetic locus controlling accumulation of fructans

Besides having potential involvement in responses to stress [20, 35, 36], fructose is an important carbohydrate in perennial ryegrass. Indeed, high-sugar varieties (with elevated levels of glucose, fructose and sucrose) have been produced through traditional breeding and were shown to improve milk and meat production whilst decreasing nitrogen losses in the waste products [1]. Thus, increased water-soluble content has been a high-priority trait in numerous breeding programmes. To search for molecular markers, a QTL analysis was utilised by [8] to dissect the genetic regulation of these traits and described several loci regulating the levels of several carbohydrates such as glucose, fructose, sucrose and polymeric fructans. Our study also describes a QTL for the levels of glucose (LG7) and fructose (LG3 and LG7), the crucial precursor for fructan biosynthesis, the major polymeric water-soluble carbohydrates in perennial ryegrass. Levels of fructose were highly correlated with glucose (0.83) and also the QTL co-localized on LG7. The study by [8] also described similar correlations (0.799–0.873) between these two metabolites and found a single QTL for fructose which co-located with a QTL for glucose. Perhaps interestingly, two further QTLs were found for glucose which did not co-locate with a fructose QTL [8]. In this study, a QTL which appeared to be specific for fructose but not glucose was observed on LG3. In future genomic selection approaches the selection of novel varieties with respect to their fructose levels without impacting on the levels of glucose or other water-soluble carbohydrates could be realized. The supplementation of fructose in the diets of dairy cattle induced with subacute acidosis was found to result in further decrease in ruminal pH by increasing lactate concentrations and increasing in the production of volatile fatty acids [37]. Higher levels of fructose could contribute towards rumen acidosis and the breeding and selection of varieties with decreased levels of fructose constitutes a potential mitigating strategy to control the prevalence of rumen acidosis in dairy cattle.

### QTLs for aminoacids

We identified QTLs for two amino-acids, L-leucine (LG1) and L-serine (LG3). Indeed, forage grasses are important dietary sources of nitrogen for ruminants which can be present in different forms such as true protein (TP), and non-protein nitrogen that includes smaller peptides, free amino acids and ammonia [38]. These experience further degradation and modification through digestion and particularly by interaction with bacterial and protozoa communities in the rumen. Indeed, there is evidence that the rumen microbial population contributes greatly to amino acid catabolism which indicates that the majority of amino acid supplementation will not survive ruminal degradation [15]. In recent years, as the number of identified hyper-ammonia-producing bacteria species have reported, it has been hypothesized that the presence of high levels of free amino acids in the diet could benefit these microbial populations [16]. Indeed, these species are highly specialized in using amino acids as their sole source of energy and cannot utilise proteins as source of energy and, as result, they are extremely efficient in deaminating amino acids leading to the production of ammonia [39]. This could subsequently impact not only amino acid bioavailability but also the levels of environmental pollution through increased elimination of ammonia in the urine. Therefore, the reduction of free amino acid levels could be a promising target for improving animal N-uptake whilst reducing the environmental impact of meat and dairy production. The findings presented in our study indicate that the levels of some free amino acids in perennial ryegrass are genetically controlled and could therefore be valid targets for the development of novel improved perennial ryegrass varieties.

### QTLs for organic acids

Citric acid is a central metabolite in the Krebs cycle and is ubiquitous in the Animal and Plant kingdoms, whereas 2, 3-dihydroxypropanoic acid is a key metabolite in the C_2_ photorespiratory cycle and is universally present in plants. The supplementation of organic acids, particularly fumarate and malate in ruminant diets has been proposed to stimulate the growth of specific ruminal microbial communities [17, 18, 40–42]. Citrate supplementation has been studied to a lesser extent but [43] indicated that supplementing diets of Chinese Simmental steers could reduce ruminal pH, increase total volatile fatty acid concentration, acetate production and digestibility. Indeed, in vitro studies have shown that citrate is rapidly metabolised by rumen bacteria [44–46] forming acetate and carbon dioxide [45]. The supplementation of citrate in ruminant diets is unlikely to ameliorate rumen acidosis, however, the improved nutrient digestibility observed in high citrate diets could contribute to improving the efficiency of feed utilisation. The inclusion of some organic acids as feed additives is unlikely to be economically feasible [17], however, optimising the levels of these compounds in the feed through selecting diets including grasses and/or grains with improved levels of organic acids remains a far more achievable strategy. The results presented in this study provide supporting evidence that levels of specific organic acids are genetically regulated and could be exploited in novel breeding programs with the aim of generating novel forage varieties that improve the efficiency of feed utilisation.

## Conclusions

We have used a targeted gas-chromatography mass spectrometry (GC-MS) approach to profile the levels of 25 polar metabolites from different classes (sugars, amino acids, phenolic acids, organic acids and other nitrogen-containing compounds) present in a large *L. perenne* F2 population consisting of 325 individuals. A high-density genetic linkage map based on 1211 GbS SNP markers from 149 genotypes from this population was used to generate initial mQTLs. By combining the SNP marker information with DArT markers from a lower-density map on the entire population and imputing missing genotypes, a more powerful QTL analysis was performed, which confirmed the initial QTLs and detected some additional smaller ones. QTL were detected that were related with carbohydrate and nitrogen metabolism. One QTL each was detected using interval mapping for L-leucine, L-serine, glucose and citric acid, whereas two QTLs each were detected for *myo*-inositol and fructose. The levels of 2,3-dihydroxypropanoic acid were found to be associated with three QTLs. The presence of these QTLs highlights the potential for improving the biochemical composition of *Lolium perenne* which can lead to downstream effects in livestock ruminal processes and productivity.

## Additional files

Additional file 1: Table S1. Selection of polar metabolites quantified using GC-MS grouped by retention index with listing of selection ions for the integration of peaks and identification based on comparison with the mass spectra of authentic standards derivatised as described in Materials and Methods. Alternatively, compounds were tentatively annotated based on comparison with the metabolomics gold standard (http://chemdata.nist.gov/dokuwiki/doku.php?id=chemdata:amdis). (DOCX 15 kb)

Additional file 2: Phenotypic metabolite data and genotypic data used for genetic map construction in .csv file in R/qtl format. (CSV 895 kb)

Additional file 3: Figure S1. Principal component analysis (PCA) based on the correlation matrix of 26 polar metabolites of parents and F1 samples of the *Lolium* mapping population. Red = P_m maternal line; blue = P_f paternal line; black = F1. (PDF 9 kb)

Additional file 4: Figure S2. Principal component analysis (PCA) based on the correlation matrix of 26 polar metabolites of parents, F1 and F2 samples of the *Lolium* mapping population. Red = P_m maternal line; blue = P_f paternal line; black = F1; green = F2. (PDF 35 kb)
