# Supplementary material for: Quantitative trait loci associated with different polar metabolites in perennial ryegrass - providing scope for breeding towards increasing certain polar metabolites
Source: BMC Genet. 2017 Oct 10;18:84. doi: 10.1186/s12863-017-0552-0 (PMC5634963; doi:10.1186/s12863-017-0552-0)
Supplement: Supplementary file 1 — Selection of polar metabolites quantified using GC-MS grouped by retention index with listing of selection ions for the integration of peaks and identification based on comparison with the mass spectra of authentic standards derivatised as described in Materials and Methods. Alternatively, compounds were tentatively annotated based on comparison with the metabolomics gold standard (http://chemdata.nist.gov/dokuwiki/doku.php?id=chemdata:amdis). (DOCX 15 kb) [file 12863_2017_552_MOESM1_ESM.docx]

**Table SI** –Selection of polar metabolites quantified using GC-MS grouped by retention index with listing of selection ions for the integration of peaks and identification based on comparison with the mass spectra of authentic standards derivatised as described in Materials and Methods. Alternatively, compounds were tentatively annotated based on comparison with the GC-MS database NIST/EPA/NIH mass spectral library (NIST 08).

| Compound Id | Retention index | m/z | Identification |
| --- | --- | --- | --- |
| L-glycine | 1300 | 174.00 | W328707 (Aldrich) |
| 2,3-dihydroxypropanoic acid | 1333 | 189.00; 292.00 | Comparison with NIST database |
| L-serine | 1366 | 204.00 | 84959 (Sigma) |
| L-threonine | 1393 | 218.50 | 89179 (Sigma) |
| Malic acid | 1499 | 233.00 | 94916 (Fluka) |
| L-aspartate | 1527 | 232.00 | 11189 (Sigma) |
| γ-aminobutyric acid | 1535 | 174.00 | A2129 (Sigma) |
| Threonic acid | 1562 | 292.00 | 380644 (Sigma) |
| L-glutamate | 1618 | 246.00 | W328502 (Aldrich) |
| Putrescine | 1742 | 174.00 | Comparison with NIST database |
| Shikimic acid | 1821 | 255.00; 357.00; 372.00; 462.00 | Comparison with NIST database |
| Citric acid | 1824 | 273.00; 363.00; 465.00 | 251275 (Sigma-Aldrich) |
| Galactose | 1891 | 319.00 | G0750 (Sigma-Aldrich) |
| L-lysine | 1923 | 174.00 | 23128 (Fluka) |
| L-tyrosine | 1939 | 218.00 | 93829 (Sigma) |
| *myo*-inositol | 2086 | 217.00; 305.00 | Comparison with NIST database |
| Glucose/Galactose-glycerol conjugate | 2309 | 204.00; 337.00 | Comparison with NIST database |
| Sucrose | 2637 | 217.00; 361.00 | S9378 (Sigma) |
| Chlorogenic acid | 3107 | 255.00; 345.00 | C3878 (Aldrich) |
| Fructose | 1873/1880 | 217.10 | F0127 (Sigma) |
| Glucose | 1896/1912 | 319.11 | G8270 (Sigma) |

*m/z*: mass:charge ratio
