# Supplementary figures and images for: Quantitative trait loci associated with different polar metabolites in perennial ryegrass - providing scope for breeding towards increasing certain polar metabolites
Source: BMC Genet. 2017 Oct 10;18:84. doi: 10.1186/s12863-017-0552-0 (PMC5634963; doi:10.1186/s12863-017-0552-0)

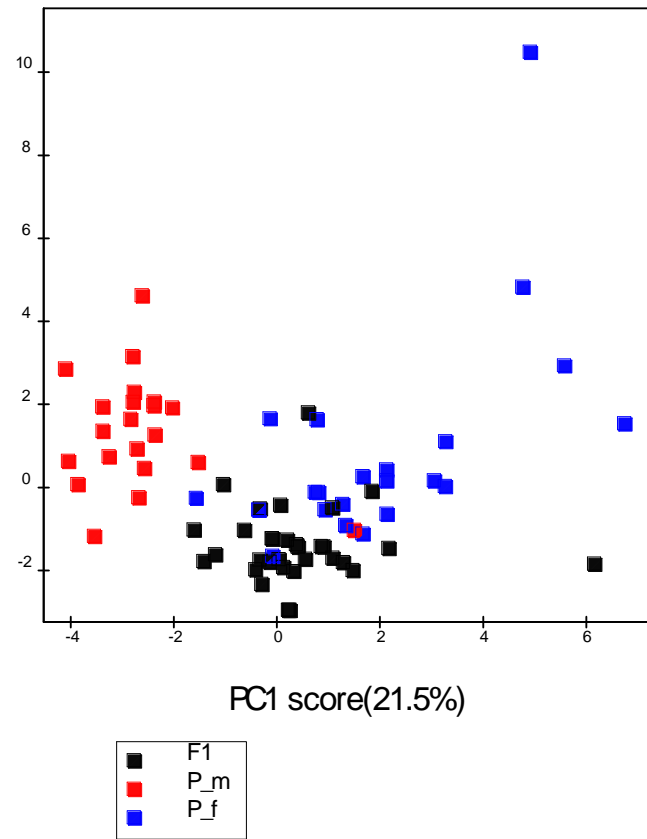

Supplement: Supplementary file 3 — Principal component analysis (PCA) based on the correlation matrix of 26 polar metabolites of parents and F1 samples of the Lolium mapping population. Red = P_m maternal line; blue = P_f paternal line; black = F1. (PDF 9 kb) [file 12863_2017_552_MOESM3_ESM.pdf]

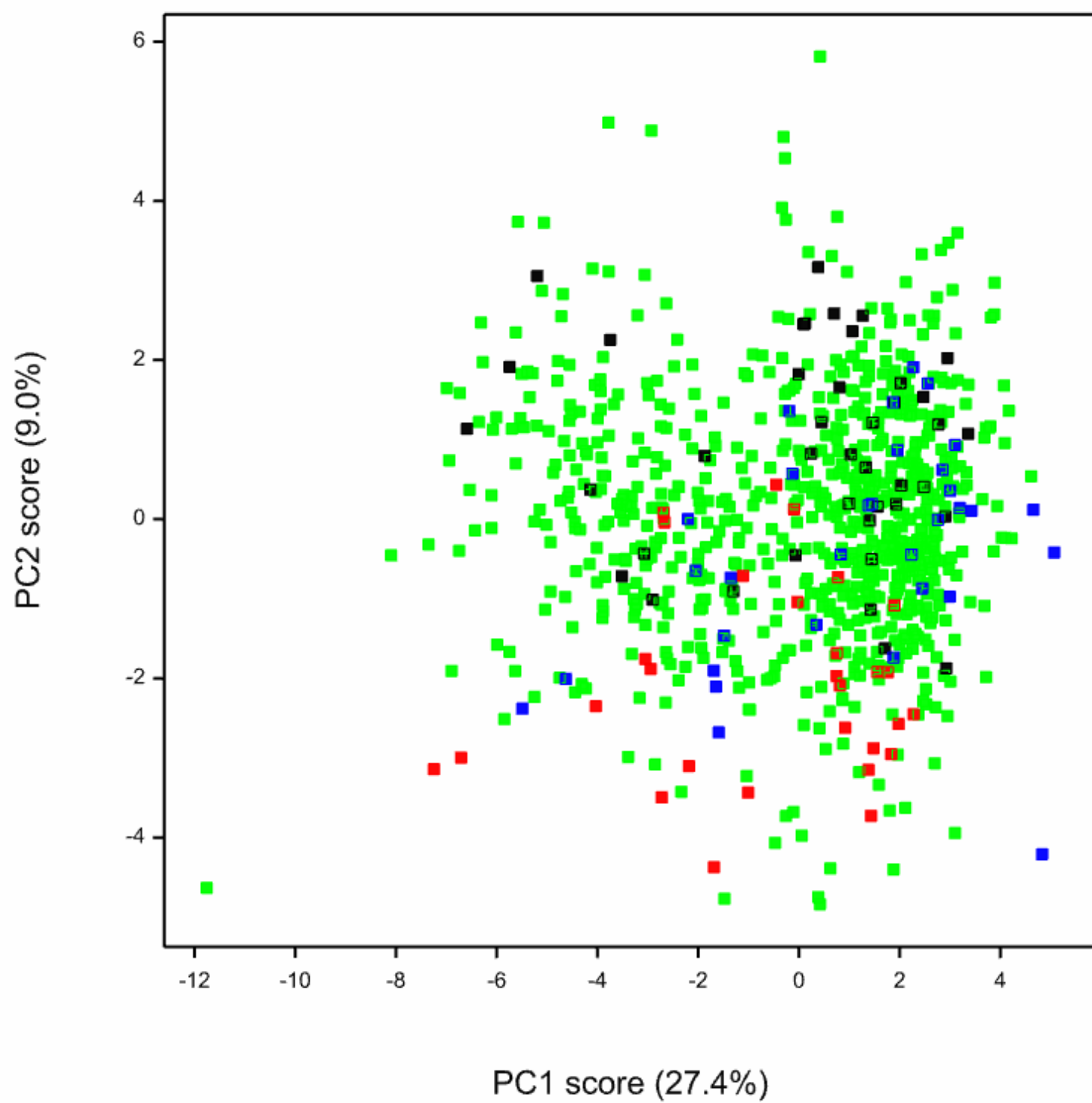

Supplement: Supplementary file 4 — Principal component analysis (PCA) based on the correlation matrix of 26 polar metabolites of parents, F1 and F2 samples of the Lolium mapping population. Red = P_m maternal line; blue = P_f paternal line; black = F1; green = F2. (PDF 35 kb) [file 12863_2017_552_MOESM4_ESM.pdf]
